# Comparative transcriptomics of spotted seatrout (*Cynoscion nebulosus*) populations to cold and heat stress

**DOI:** 10.1002/ece3.7138

**Published:** 2020-12-28

**Authors:** Jingwei Song, Jan R. McDowell

**Affiliations:** ^1^ Virginia Institute of Marine Science (VIMS) College of William and Mary Gloucester Point VA USA

**Keywords:** climate change, *Cynoscion nebulosus*, phenotypic plasticity, RNA‐seq, temperature stress, transcriptome

## Abstract

Resilience to climate change depends on a species' adaptive potential and phenotypic plasticity. The latter can enhance survival of individual organisms during short periods of extreme environmental perturbations, allowing genetic adaptation to take place over generations. Along the U.S. East Coast, estuarine‐dependent spotted seatrout (*Cynoscion nebulosus*) populations span a steep temperature gradient that provides an ideal opportunity to explore the molecular basis of phenotypic plasticity. Genetically distinct spotted seatrout sampled from a northern and a southern population were exposed to acute cold and heat stress (5 biological replicates in each treatment and control group), and their transcriptomic responses were compared using RNA‐sequencing (RNA‐seq). The southern population showed a larger transcriptomic response to acute cold stress, whereas the northern population showed a larger transcriptomic response to acute heat stress compared with their respective population controls. Shared transcripts showing significant differences in expression levels were predominantly enriched in pathways that included metabolism, transcriptional regulation, and immune response. In response to heat stress, only the northern population significantly upregulated genes in the apoptosis pathway, which could suggest greater vulnerability to future heat waves in this population as compared to the southern population. Genes showing population‐specific patterns of expression, including *hpt*, *acot*, *hspa5*, and *hsc71*, are candidates for future studies aiming to monitor intraspecific differences in temperature stress responses in spotted seatrout. Our findings contribute to the current understanding of phenotypic plasticity and provide a basis for predicting the response of a eurythermal fish species to future extreme temperatures.

## Graphical abstract

Spotted seatrout, *Cynoscion nebulosus*, is a popular saltwater sport fish in the United States, and distinct populations exist along the US east coast. We *de novo* assembled the first high‐quality liver transcriptome of the species and discovered both shared and unique transcriptomic responses after cold and heat stress in fish with distinct genetic background.

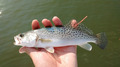

## 1 INTRODUCTION

Temperature has direct and pervasive effects on fish physiology (Angilletta et al., 2010; Fry, 1947). Since most fish are ectothermic and their body temperature tracks ambient water temperature, temperature governs the rate of biochemical reactions (Allen et al., 2002), metabolic rates (Chabot et al., 2016), and ultimately the distribution of species (Pinsky et al., 2013). There is ample evidence that fish populations from contrasting thermal regimes show divergent physiological responses to water temperatures, likely due to local adaptation. For example, sockeye salmon (*Oncorhynchus nerka*) populations were found to differ in their cardiovascular physiology, which correlated with the historical river temperatures each population encountered during upriver migration (Eliason et al., 2011). Common killifish (*Fundulus heteroclitus*) collected from their northern and southern range limit along the western Atlantic coast show different thermal tolerance; the critical thermal maximum was significantly higher in the southern population (~1.5°C; Fangue, 2006). The underlying molecular mechanisms, however, are complex and only starting to be explored for fishes (Oomen & Hutchings, 2017). This line of research has historically been difficult as quantifying gene expression in nonmodel species was limited to a few candidate genes or required significant investment of time and expertise to generate species‐specific resources (e.g., microarrays).

Next‐generation sequencing and RNA‐sequencing (RNA‐seq) have become more accessible and allowed the transcriptome (whole set of the messenger RNA molecules in a cell or tissue) of any organisms to be studied (Alvarez et al., 2015; Todd et al., 2016). Previous studies looking at gene expression differences have generally focused on sublethal temperature stress lasting from weeks to months (Narum & Campbell, 2015; Newton et al., 2013; Veilleux et al., 2018). How gene expression responds to acute temperature stress on the scale of hours to days is less well understood (Buckley, 2006; Healy et al., 2010). It is predicted that extreme episodes of water temperature change, such as marine heat waves, will increase in frequency due to climate change (Trenberth & Fasullo, 2012). Transcriptomic studies can improve our understanding of the molecular mechanisms underlying these short‐term events and help predict which population might be more vulnerable to these events.

Spotted seatrout, *Cynoscion nebulosus*, is a teleostean fish distributed from coastal waters in New York to the Gulf of Mexico (Bortone, 2002). This species is uncommon north of Chesapeake Bay, most likely due to its low survival in water temperatures below 5°C (Ellis et al., 2017). Spotted seatrout is comprised of several genetically distinct populations throughout its geographic range (Anderson & Karel, 2009; Seyoum et al., 2018; Weinstein & Yerger, 1976), which provides an ideal opportunity to study differences in thermal plasticity among populations. Along the U.S. East Coast, spotted seatrout in Chesapeake Bay are genetically distinct from those in South Carolina and further south (McDowell et al., 2015; Wiley & Chapman, 2003). Based on genome‐wide single nucleotide polymorphism markers (Song, 2020), the level of genetic differentiation between spotted seatrout sampled from Corrotoman River, Virginia, and those sampled from James Island, South Carolina, was *F*_ST_ = 0.049, *p* < .001. Similar results were found based on microsatellite markers (Great Wicomico River, VA, and Charleston Harbor, South Carolina, *F*_ST_ = 0.043, *p* < .001; Ellis et al., 2019). Studies of spotted seatrout on the East Coast of the United States have also revealed a range of physiological and life history differences such as growth rate (Smith et al., 2008), size at maturity (Brown‐Peterson, 2003; Ihde, 2000), and metabolic rate (Song et al., 2019), but whether these populations also respond differently to thermal stress is unknown.

Fish populations that experience contrasting temperature regimes show distinct transcriptomic responses, likely as a result of local adaptation (Newton et al., 2013; Veilleux et al., 2018). Spotted seatrout living at its northern range limit in Chesapeake Bay encounter near‐freezing water temperature yearly (McGrath & Hilton, 2017), while its southern counterparts rarely experience temperatures below 5°C. In summer, however, maximum water temperature in Chesapeake Bay can reach above 30°C, which is similar to temperatures experienced by fish in more southern regions (Lewisetta, VA, and Charleston, SC, National Centers for Environmental Information, National Oceanic and Atmospheric Administration). Episodic winterkills of spotted seatrout are common in Virginia, but rare in South Carolina and further south on the US East Coast (Ellis et al., 2017). The differences in winter severity suggest that the two populations are under different selective pressure by temperature and this may have led to distinct transcriptomic responses to acute cold and heat stresses.

Previous study has shown that the northern spotted seatrout population has greater capacity for metabolic plasticity than the southern population from 5°C to 30°C (Song et al., 2019). The purpose of this study was to compare the underlying transcriptomic response to acute temperature stress in these two genetically distinct and physiologically divergent spotted seatrout populations to better understand the observed metabolic differences. The objectives were threefold: (a) to construct a high‐quality transcriptome for spotted seatrout, (b) to discover and quantify shared transcriptomic responses to cold and heat stress in both populations, and (c) to discover and quantify unique transcriptomic responses to cold and heat stress in each population.

## 2 MATERIALS AND METHODS

### 2.1 Sample collection and acclimation conditions

Adult spotted seatrout (*Cynoscion nebulosus*) were collected from the Corrotoman River, Virginia (“VA”, 37°43′58.8″N, 76°24′32.3″W), and James Island, South Carolina (“SC”, 32°45′11.0″N, 79°53′48.0″W), between 2016 and 2018 (Table 1). The F1 generation from wild SC parents was also included to supplement the sample size of the SC wild‐caught fish due to logistical difficulties with sample collection and transport (Table S1). Fish were acclimated in flow‐through 10,000‐L circular tanks with brackish water (salinity 20–22 ppt) from the York River, Virginia. Water temperature was maintained at 15°C prior to cold stress experiments and at 20°C prior to heat stress experiments. Different acclimation temperatures were used for heat and cold stress experiments to achieve the same level of temperature change (10°C decrease or increase; see next section). Cold‐stressed fish from both populations were collected and experimented upon during the fall/winter months, whereas the heat‐stressed fish were collected during the fall or spring and experimented upon during the summer. The acclimation temperatures were chosen because they represent common water temperatures that spotted seatrout commonly experience in the fall and spring, respectively. Differences in acclimation lengths (30–105 days; Table S1) were due to different sampling dates, as well as experimental dates (Song et al., 2019). Nevertheless, all fish were acclimated at least 30 days prior to cold and heat stress. All fish were fed frozen and thawed bay anchovies (*Anchoa mitchilli*) every 2 days to satiation.

**TABLE 1:** Spotted seatrout samples used in this study

Treatment group	Sample ID	Sampling temperature (°C)	Sample size
SC cold	SC_c_1 to SC_c_5	5	5
VA cold	VA_c_1 to VA_c_5	5	5
SC heat	SC_h_1 to SC_h_5	30	5
VA heat	VA_h_1 to VA_h_5	30	5
SC control	SC_ctrl_1 to SC_ctrl_5	15	5
VA control	VA_ctrl_1 to VA_ctrl_5	15	5

Abbreviations: SC, James Island, South Carolina; VA, Corrotoman River, Virginia.

### 2.2 Experimental design

Experimental fish were held in cylindrical respirometers immersed in water as part of a previous study measuring metabolic rates of spotted seatrout at various temperatures (Song et al., 2019). Briefly, fish from both VA and SC in the cold stress group were consecutively exposed to decreasing temperatures starting from the acclimation temperature at 15°C (20 hr), 10°C (5 hr), and 5°C (18 hr) at a rate of −2.5°C/hr. The rate of temperature decrease was chosen because winterkills of spotted seatrout typically involve rapid temperature decreases due to cold fronts or snow melt (Ellis et al., 2017). Fish in the heat stress group were exposed to increasing water temperatures: 20°C (20 hr), 25°C (5 hr), and 30°C (18 hr) at a rate of 2.5°C/hr. This rate of temperature increase was chosen to mirror the cold shock experiment and to simulate potential heat shocks in the shallow estuarine habitats for spotted seatrout. At the end of each respirometry experiment, fish were euthanized by applying cranial concussion followed by pithing. This 2‐step protocol follows the American Veterinary Medical Association Guideline for the Euthanasia of Animals (Leary & Johnson, 2020). This protocol is faster than other euthanasia methods, such as immersing fish in icy water mix or using tricaine methanesulfonate (MS‐222), and therefore minimized the impact of euthanasia on gene expression. A small piece of liver tissue from each fish was collected after euthanasia using sterilized scissors. Liver tissue was used in this study because it is a key regulator for metabolic processes and produces many stress‐responsive proteins (Currie & Schulte, 2013). The tissues were stored in cryovials and flash‐frozen in liquid nitrogen until RNA extraction. Spotted seatrout in the control groups were directly netted out of the holding tanks (15°C), and liver tissues were preserved as described above. All animal care and use protocols were approved by William & Mary's Institutional Animal Care and Use Committee (IACUC‐2017‐09‐25‐12356‐jrmcdo).

### 2.3 RNA extraction and RNA‐seq

Liver tissue (~20 mg) from each sample was weighed and pulverized in liquid nitrogen using a mortar and pestle. The mortar and pestle were thoroughly cleaned by rinsing under deionized water and wiped with RNase AWAY solution (Thermo Scientific) between samples to ensure no RNA contamination occurred. Total RNA was extracted from the pulverized tissues using the RNeasy Mini Kit (Qiagen) according to the manufacturer's protocol including the on‐column DNase digestion step to eliminate genomic DNA. RNA concentration was determined using a Qubit 2.0 fluorometer (BR RNA Assay, Invitrogen). RNA quality was assessed in two ways. First, the ratio of absorbance at 260 and 280 nm (260/280) was evaluated using a NanoDrop ND‐1000 spectrophotometer (NanoDrop Technologies). Samples with a 260/280 around 2 suggest high purity of RNA. Second, a 5 µl aliquot of each sample was loaded onto a 1% agarose gel mixed with GelRed (Biotium), immersed in 1× Tris/Borate/EDTA (TBE) buffer, electrophoresed at 100 V for 35 min, and visualized under UV transillumination. The presence of two bright bands representing the 28s and 18s rRNA was used to evaluate intactness of the RNA. All samples met the criteria above. Samples of extracted RNA were then shipped on dry ice to the Biocomplexity Institute (BI) at Virginia Polytechnic Institute and State University. The quality and quantity of the RNA samples were again assessed using a TapeStation (Agilent Technologies). RNA samples were standardized to 50 ng/µl, and a fresh 25 µl aliquot of each RNA sample was sent in a second shipment to BI for construction of cDNA libraries using an Illumina TruSeq Stranded mRNA HT Sample Prep Kit (Illumina). The resulting libraries were multiplexed and paired‐end‐sequenced using a NextSeq 500/550 High Output kit V2 (2× 75 bp cycles, 400 million clusters). The Illumina NextSeq Control Software v2.1.0.32 with Real‐Time Analysis RTA v2.4.11.0 was used to provide the management and execution of the NextSeq 500 sequencing run and to generate binary base call (BCL) files. The BCL files were converted to FASTQ files, adapters were trimmed, and reads were demultiplexed using bcl2fastq Conversion Software v2.20 (Illumina). Raw data were submitted to NCBI's short read archive (accession PRJNA649515, release date 2021‐08‐07).

### 2.4 Bioinformatic analyses

Adapted–trimmed FASTQ files were downloaded to Carbonate (https://kb.iu.edu/d/aopq), Indiana University's large‐memory computer cluster. For each sample, there was a FASTQ file containing all the forward sequencing reads and a FASTQ file containing all the reverse sequencing reads. FastQC (v 0.11.8) was used to check the quality scores across all bases (Andrews, 2015). To reduce the quantity of the input reads for de novo assembly and to improve assembly efficiency (Brown et al., 2012; Fletcher et al., 2013), forward and reverse reads from each sample were first concatenated and in silico normalization was performed using the default setting in Trinity v2.8.4 (Haas et al., 2013). Different assembly algorithms can complement each other in discovering genes that might be missed using a single method (MacManes, 2018); therefore, de novo transcriptome assembly was performed on the in silico*‐*normalized data using four different programs with varying k‐mer sizes (subsequences with length k within a longer sequence): Trinity v2.8.4 (k‐mer = 25; Haas et al., 2013), Velvet‐Oases v1.2.10 (k‐mer = 35, 45, 55; Schulz et al., 2012), SOAPdenovo v1.03 (k‐mer = 35, 45, 55; Xie et al., 2014), and TransAbyss v1.5.5 (k‐mer = 35, 45, 55; Robertson et al., 2010). All assemblies were concatenated into a single transcriptome and further processed with the EvidentialGene tr2aacds pipeline to select high‐quality sequences based on length and gene‐coding potential and to reduce redundancy (Evigene, http://eugenes.org/EvidentialGene; Gilbert, 2013). This resulted in a final version of the spotted seatrout liver transcriptome (File S1, accessible on Dryad, https://doi.org/10.5061/dryad.3j9kd51dx).

To assess the quality of the newly generated spotted seatrout transcriptome, QUAST v4.6.3 (Gurevich et al., 2013) was used to calculate common genomic metrics, including transcript length summary, N50 (length of at least half of all the transcripts) and GC content. BUSCO v3.0.2 (Benchmarking Universal Single‐Copy Orthologs) was used to assess the completeness of the assembled transcriptome in terms of expected gene content (Simão et al., 2015). Specifically, the spotted seatrout liver transcriptome was searched against the database actinopterygii_odb9, which contains 4,584 evolutionarily conserved genes expected to be found as single‐copy orthologs in at least 90% of the Actinopterygii (ray‐finned fishes).

To assess gene expression levels for each transcript in the transcriptome for all 30 fish, original sequences were mapped back to the new transcriptome using kallisto v0.43.1 (Bray et al., 2016). Kallisto uses a novel “pseudoalignment” approach to eliminate the need for perfectly aligning individual bases, thereby reducing the computing time by two orders of magnitude compared with alternative programs while achieving similar mapping accuracy (Bray et al., 2016). Transcript abundances were normalized and reported in transcripts per million (TPM).

To assess differential expression, two programs with different statistical frameworks were used: DESeq2 (Love et al., 2014) and limma + voom (Law et al., 2014; Ritchie et al., 2015). Currently, there is no clear consensus on which differential expression algorithm achieves the best balance between type I and type II errors (Costa‐Silva et al., 2017; Soneson & Delorenzi, 2013). Similar to our use of different de novo assemblers, we used more than one approach to detect consensus transcripts because a false positive is less likely to be identified twice. DESeq2 uses a negative binomial distribution and a shrinkage estimator for dispersion and fold change. Limma + voom uses normal distribution and fits a linear model with all genes and samples combined. Both methods have been shown to achieve a good balance between accuracy and sensitivity when the number of biological replicates is at least three (five biological replicates in this study; Costa‐Silva et al., 2017). Contrasts were done as follows: VA cold versus VA control, VA heat versus VA control, SC cold versus SC control, and SC heat versus SC control. Differentially expressed genes (DEGs) were defined as those transcripts with an absolute log2 fold change greater than two and a multitest adjusted *p* < .05 (Benjamini & Hochberg, 1995). DEGs identified by both methods were retained and used in downstream analyses.

The new transcriptome was annotated using Trinotate v3.1.1 (Bryant et al., 2017), which conducts BLASTx and BLASTp searches against the Swiss‐Prot database to identify matches to known proteins using default E‐value cutoffs (Altschul et al., 1990; The Uniprot Consortium, 2019). Based on the matches from both BLASTx and BLASTp searches, Trinotate extracts Gene Ontology (GO) (The Gene Ontology Consortium, 2019) terms and K numbers from the Kyoto Encyclopedia of Genes and Genomes (KEGG) Orthology Database (Kanehisa et al., 2016). The results from Trinotate were stored in a tab‐delimited file (File S2). Higher‐level molecular pathways were discovered based on the K numbers associated with differentially expressed transcripts for both cold and heat stress in each population (Kanehisa & Sato, 2019). The search mode was set to spotted seatrout's closest relative available in the database: large yellow croaker, *Larimichthys crocea* (lco). To assess the presence of significantly enriched GO biological processes, the R package topGO v. 2.38.1 (Alexa et al., 2006) was used with the classic Fisher's exact test, *p* < .01. All GO terms found using Trinotate were used as the gene universe (“reference”) and DEGs were used as target lists (“interesting genes”).

### 2.5 Validation using RT‐qPCR

To validate the accuracy of RNA‐seq results, real‐time quantitative polymerase chain reaction (RT‐qPCR) was used to calculate log_2_ fold change of a subset of the transcripts (*n* = 6). Primers were designed based on transcripts assembled in the de novo transcriptome using Primer‐BLAST (Ye et al., 2012), with primer length set to 20 bp and melting temperature set to 60°C (Table S2). 18S ribosomal RNA was used as an internal control using primers from Brewton et al., 2013, and VA and SC control group samples were used as reference samples. Total RNA (1 µg) was reverse‐transcribed using the SuperScript IV First‐Strand Synthesis System (Invitrogen) with both the random hexamers (50 ng/µl) and oligo(dT)_20_ (50 µM), and synthesized cDNA template was diluted 1:100. Each 20 µl reaction consisted of 10 µl of PowerUp™ SYBR™ Green Master Mix (2×), 1 µl for each of the forward and reverse primers (10 µM), 1 µl of cDNA template, and 7 µl of PCR‐grade water. All reactions were run on a QuantStudio 3 Real‐Time PCR System (Thermo Fisher Scientific). Thermocycling conditions were as follows: 50°C (2 min), 95°C (10 min), 40 cycles of 95°C (15 s), and 60°C (1 min). Melt curve analyses were conducted immediately following thermal cycling with 95°C (15 s), 60°C (1 min), and 95°C (15 s). Each reaction was performed in triplicate, and the mean threshold cycle (C_T_) was used for subsequent analyses. The comparative C_T_ method was used to present log_2_ fold change in order to make results directly comparable to RNA‐seq results. To assess the validity of using the comparative C_T_ method, PCR efficiencies of all primers were calculated by running a standard curve with five 1:10 serial dilution points (Schmittgen & Livak, 2008).

## 3 RESULTS

### 3.1 Transcriptome assembly

**FIGURE 1:**
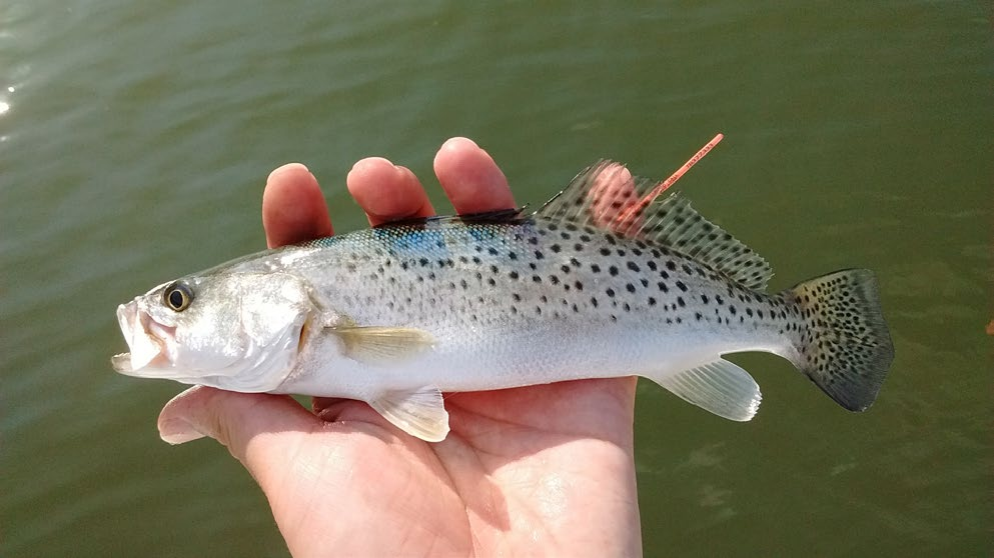
Spotted seatrout, *Cynoscion nebulosus*, on York River, Virginia. Photo by Jingwei Song

All 30 samples had an RNA Integration Number (RIN) value greater than 8, indicating high‐quality RNA (Figure 1). Sequencing reads per sample ranged from 13.6 million to 18.6 million (mean ± *SD* = 15.7 ± 1.2), and visual examination of fastQC reports confirmed good average quality scores across all bases (average quality score > 28). After in silico normalization, 6.6 million paired‐end reads were retained for de novo transcriptome assembly. Four different assemblers produced the following numbers of transcripts with the results from different k‐mers combined: Trinity, 185,556; SOAPdenovo, 403,848; TransAbyss, 209,799; and Velvet, 416,310. The final spotted seatrout liver transcriptome consisted of 37,398 transcripts.

The quality of the de novo‐assembled spotted seatrout liver transcriptome was assessed using QUAST and BUSCO. QUAST indicated that this transcriptome contained 21,316 transcripts longer than 500 bp, an N50 of 3,121 bp, and a GC content of 48.98% (Table S3). BUSCO analysis found that this transcriptome assembly contained 81.3% complete gene sequence information and 6.1% fragmented BUSCOs of the essential genes in bony fish genomes (lineage dataset: actinopterygii_odb9, 20 species, 4,584 total BUSCO groups searched).

### 3.2 Gene expression plasticity among and within populations

**FIGURE 2:**
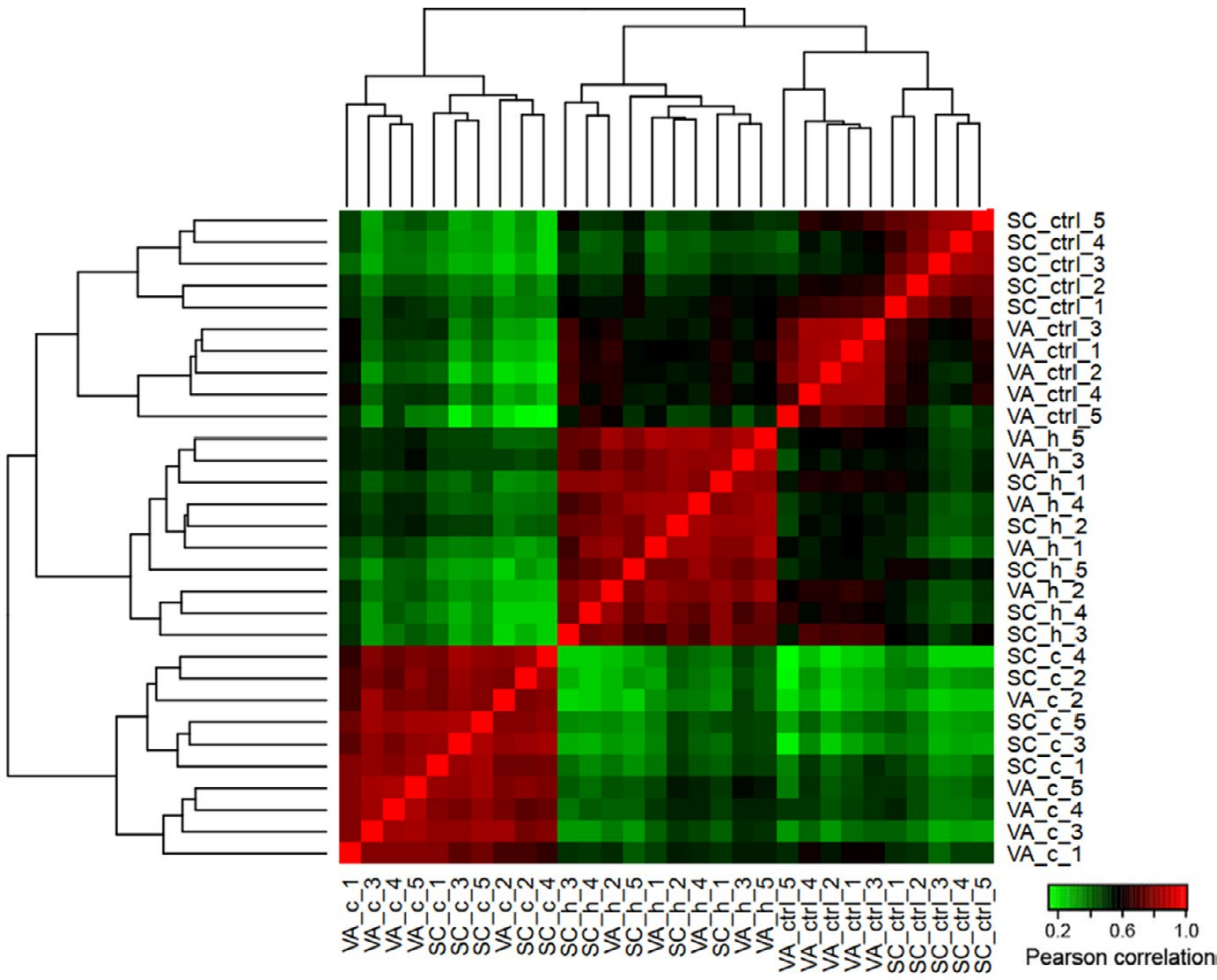
Heatmap showing pairwise Pearson's correlation values of gene expression for all 30 spotted seatrout RNA‐seq samples. c, cold stress; h, heat stress; ctrl, control; SC, southern population; VA, northern population

Gene expression levels showed good correlation among biological replicates within treatment and control groups (Pearson's *r* = .89, Figure 2). While the two populations clearly separated in the control groups, they did not form distinct clusters in heat and cold treatment groups (Figure 2, Figure S1). A total of 1,653 DEGs were found in VA and SC samples combined in response to cold and heat stress treatments. Of these, 1,281 DEGs were found in the cold treatment groups, and 570 DEGs were found in the heat treatment groups as compared to controls. A set of 20 DEGs were responsive to both heat and cold stress in both populations (File S3, Figure S2).

**FIGURE 3:**
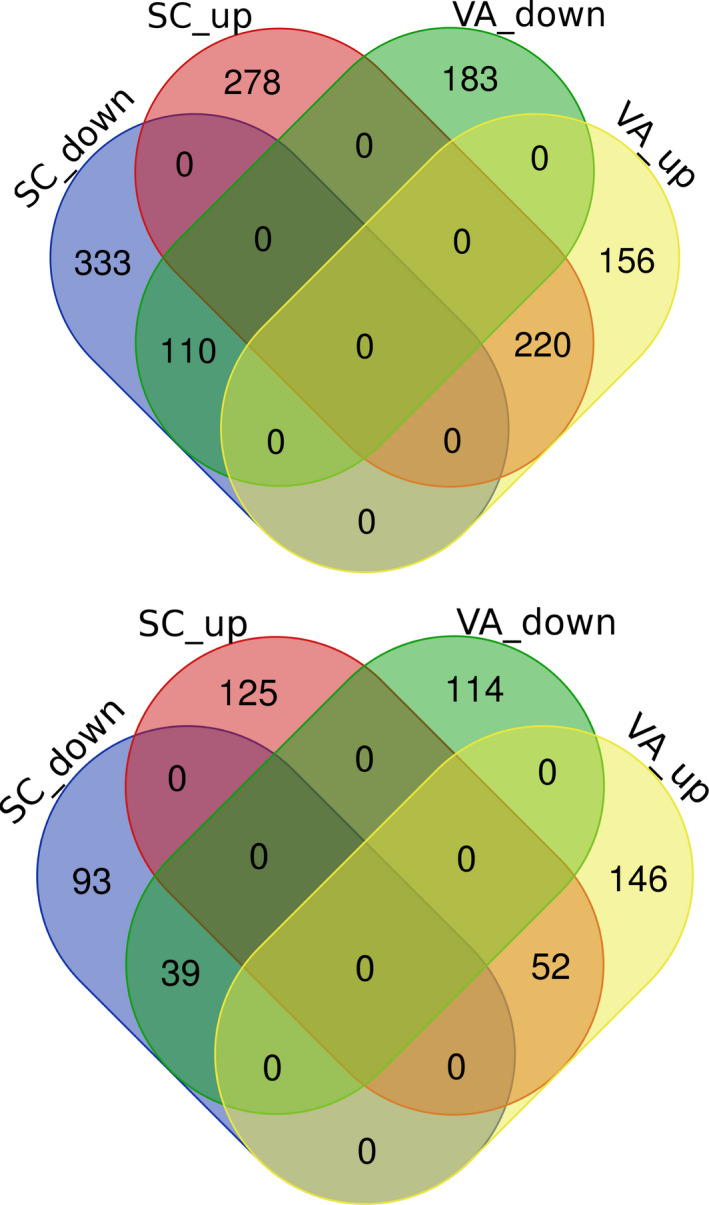
Venn diagram showing the number of differentially expressed genes (DEGs) between the northern (VA) and southern (SC) spotted seatrout populations. Top: cold stress, bottom: heat stress

Comparing the number of DEGs by population, 40% more DEGs were found in SC (941) as compared to VA (669) in response to cold stress. In response to heat stress, 14% more DEGs were found in VA (351) as compared to SC (309) (Figure 3a,b). In cold stress treatments, VA spotted seatrout had 376 upregulated versus 293 downregulated transcripts, while SC spotted seatrout had 498 upregulated transcripts versus 443 downregulated transcripts. In the heat stress treatment, VA spotted seatrout had 198 upregulated transcripts versus 153 downregulated transcripts, and SC spotted seatrout had 177 upregulated transcripts versus 132 downregulated transcripts. A complete list of all the significant DEGs and the matrix of transcript quantification can be found in the supplemental information (Files S4 and S5).

### 3.3 Functional analysis

Among the 37,398 transcripts in the transcriptome, Trinotate found 12,778 unique BLASTx hits and 12,594 unique BLASTp matches. Within the BLASTp hits, 7,715 transcripts were matched across at least 80% of the length of the target proteins. Trinotate retrieved 7,027 K numbers from the KEGG Orthology Database and 18,323 GO terms.

Based on the K numbers associated with DEGs, four lists of higher‐level molecular pathways were obtained (2 populations × 2 temperature stress treatments). In the VA cold stress group, a total of 104 pathways were discovered based on 117 K numbers. Top pathways included metabolic (lco01100), apoptosis (lco04210), insulin signaling (lco04910), forkhead box O (FoxO) signaling (lco04068), and protein processing in endoplasmic reticulum (lco04141) pathways (Table 2). A total of 120 pathways were discovered based on 213 K numbers in SC spotted seatrout subjected to cold stress. Top pathways included metabolic (lco01100), mitogen‐activated protein kinase (MAPK) signaling (lco04010), apelin signaling (lco04371), FoxO signaling (lco04068), and steroid biosynthesis (lco00100) pathways. There were 91 common cold stress pathways, with 13 unique pathways in VA spotted seatrout and 29 unique pathways in SC spotted seatrout.

**TABLE 2:** Top 10 KEGG pathways in both northern (VA) and southern (SC) spotted seatrout populations to acute temperature stress

KEGG	VA	KEGG	SC
**Cold stress**			
lco01100	*Metabolic pathways (46)*	lco01100	*Metabolic pathways (69)*
lco04210	*Apoptosis (9)*	lco04010	*MAPK signaling pathway (11)*
lco04910	*Insulin signaling pathway (9)*	lco04371	*Apelin signaling pathway (9)*
lco04068	*FoxO signaling pathway (9)*	lco04068	*FoxO signaling pathway (8)*
lco04141	Protein processing in endoplasmic reticulum (9)	lco00100	Steroid biosynthesis (8)
lco04010	*MAPK signaling pathway (8)*	lco04920	Adipocytokine signaling pathway (7)
lco00561	Glycerolipid metabolism (7)	lco04210	*Apoptosis (7)*
lco04371	*Apelin signaling pathway (7)*	lco03320	PPAR signaling pathway (7)
lco04530	*Tight junction (6)*	lco04910	*Insulin signaling pathway (6)*
lco04110	Cell cycle (6)	lco04530	*Tight junction (6)*
**Heat stress**			
lco01100	*Metabolic pathways (29)*	lco01100	*Metabolic pathways (24)*
lco04141	*Protein processing in endoplasmic reticulum (13)*	lco04010	*MAPK signaling pathway (5)*
lco00510	*N*‐Glycan biosynthesis (5)	lco04920	Adipocytokine signaling pathway (4)
lco04010	*MAPK signaling pathway (4)*	lco03320	PPAR signaling pathway (4)
lco00230	Purine metabolism (4)	lco04218	Cellular senescence (4)
lco00564	Glycerophospholipid metabolism (4)	lco04514	Cell adhesion molecules (CAMs) (3)
lco04210	Apoptosis (3)	lco00071	Fatty acid degradation (3)
lco01230	Biosynthesis of amino acids (3)	lco01212	Fatty acid metabolism (3)
lco04060	Cytokine‐cytokine receptor interaction (3)	lco04141	*Protein processing in endoplasmic reticulum (3)*
lco04625	C‐type lectin receptor signaling pathway (4)	lco04310	Wnt signaling pathway (3)

Note

Numbers in the parentheses indicate KEGG Orthology terms identified in that specific pathway. Italicized pathway names are common between the two populations.

In the heat stress group, a total of 82 KEGG pathways were discovered based on 102 K numbers in the VA spotted seatrout. Top pathways included metabolic (lco01100), protein processing in endoplasmic reticulum (lco04141), *N*‐glycan biosynthesis (lco00510), MAPK signaling (lco04010), and purine metabolism (lco00230) pathways (Table 2). A total of 71 KEGG pathways were discovered based on 79 K numbers in the SC heat stress group. Top pathways included metabolic (lco01100), MAPK signaling (lco04010), adipocytokine signaling (lco04920), PPAR signaling (lco03320), and cellular senescence (lco04218) pathways. There were 53 common pathways with 29 unique pathways in VA and 18 unique pathways in SC. A list of all the molecular pathways can be found in the supplemental information (File S6).

GO enrichment analyses revealed common and unique biological pathways between populations in response to thermal stress (Table 3). Upregulation in response to cold stress in both populations was generally related to lipid storage (e.g., GO:0050873 brown fat cell differentiation, GO:0010889 regulation of sequestering of triglyceride), cell differentiation (GO:0045647, negative regulation of erythrocyte differentiation), and protein activation cascade (e.g., GO:0072376 protein activation cascade, GO:0000185 activation of MAPKKK activity). Downregulated processes included metabolic processes (e.g., GO:0016126 sterol biosynthetic process, GO:000695 cholesterol biosynthetic process, small molecule metabolic process). In addition, VA fish were enriched in GO terms related to transport (e.g., GO:0003333 amino acid transmembrane transport, GO:0072348 sulfur compound transport).

**TABLE 3:** Top significantly enriched biological processes based on Gene Ontology analysis by population and direction of gene expression change: top: heat stress, bottom: cold stress

	SC			VA		
	GO.ID	Term	*p*‐value	GO.ID	Term	*p*‐value
**Heat**						
Upregulation	GO:0072376	Protein activation cascade	8.40E‐10	GO:1901566	Organonitrogen compound biosynthetic process	4.50E‐08
	GO:0006956	Complement activation	4.00E‐09	GO:0006457	Protein folding	6.00E‐06
	GO:0006957	Complement activation, alternative pathway	2.80E‐08	GO:0006487	Protein *N*‐linked glycosylation	2.00E‐05
	GO:0006959	Humoral immune response	1.50E‐07	GO:0009101	Glycoprotein biosynthetic process	2.40E‐05
	GO:0006082	Organic acid metabolic process	5.70E‐07	GO:1901137	Carbohydrate derivative biosynthetic process	4.30E‐05
	GO:0044281	Small molecule metabolic process	1.80E‐06	GO:0044281	Small molecule metabolic process	4.70E‐05
	GO:0002526	Acute inflammatory response	2.30E‐06	GO:0009100	Glycoprotein metabolic process	9.90E‐05
	GO:0044282	Small molecule catabolic process	2.60E‐06	GO:0006979	Response to oxidative stress	0.00012
	GO:1901605	Alpha‐amino acid metabolic process	3.10E‐06	GO:0002292	T cell differentiation involved in immune response	0.00012
	GO:0019752	Carboxylic acid metabolic process	3.10E‐06	GO:0010878	Cholesterol storage	0.00025
Downregulation	GO:0006003	Fructose 2,6‐bisphosphate metabolic process	4.00E‐06	GO:0003333	Amino acid transmembrane transport	4.10E‐07
	GO:0006000	Fructose metabolic process	1.10E‐05	GO:0072348	Sulfur compound transport	3.30E‐06
	GO:0062013	Positive regulation of small molecule metabolic process	0.00019	GO:1903825	Organic acid transmembrane transport	1.30E‐05
	GO:0044262	Cellular carbohydrate metabolic process	0.00075	GO:1905039	Carboxylic acid transmembrane transport	1.30E‐05
	GO:0042159	Lipoprotein catabolic process	0.00077	GO:0006865	Amino acid transport	7.60E‐05
	GO:0045913	Positive regulation of carbohydrate metabolic process	0.00079	GO:0046394	Carboxylic acid biosynthetic process	8.10E‐05
	GO:0030813	Positive regulation of nucleotide catabolic process	0.00101	GO:0016053	Organic acid biosynthetic process	8.50E‐05
	GO:0045821	Positive regulation of glycolytic process	0.00101	GO:0098656	Anion transmembrane transport	0.00012
	GO:0051197	Positive regulation of coenzyme metabolic process	0.00101	GO:0072330	Monocarboxylic acid biosynthetic process	0.00047
	GO:0005975	Carbohydrate metabolic process	0.00141	GO:0006003	Fructose 2,6‐bisphosphate metabolic process	0.00073
**Cold**						
Upregulation	GO:0048545	Response to steroid hormone	5.60E‐08	GO:0045647	Negative regulation of erythrocyte differentiation	7.10E‐06
	GO:0033993	Response to lipid	7.60E‐06	GO:1905952	Regulation of lipid localization	8.30E‐06
	GO:0050896	Response to stimulus	8.00E‐06	GO:0051348	Negative regulation of transferase activity	1.10E‐05
	GO:0045647	Negative regulation of erythrocyte differentiation	1.20E‐05	GO:0010889	Regulation of sequestering of triglyceride	1.40E‐05
	GO:0050873	Brown fat cell differentiation	1.20E‐05	GO:0098656	Anion transmembrane transport	1.70E‐05
	GO:0006950	Response to stress	1.30E‐05	GO:0030730	Sequestering of triglyceride	2.50E‐05
	GO:0072376	Protein activation cascade	1.40E‐05	GO:0033673	Negative regulation of kinase activity	8.20E‐05
	GO:0044283	Small molecule biosynthetic process	1.60E‐05	GO:0045638	Negative regulation of myeloid cell differentiation	0.00012
	GO:0009605	Response to external stimulus	2.10E‐05	GO:0006457	Protein folding	0.00015
	GO:0010889	Regulation of sequestering of triglyceride	2.30E‐05	GO:0000185	Activation of MAPKKK activity	0.00016
Downregulation	GO:0016126	Sterol biosynthetic process	8.60E‐13	GO:0044281	Small molecule metabolic process	1.30E‐09
	GO:0006695	Cholesterol biosynthetic process	8.90E‐11	GO:0019752	Carboxylic acid metabolic process	6.90E‐09
	GO:1902653	Secondary alcohol biosynthetic process	8.90E‐11	GO:0032787	Monocarboxylic acid metabolic process	2.30E‐08
	GO:0006694	Steroid biosynthetic process	1.10E‐10	GO:0043436	Oxoacid metabolic process	2.70E‐08
	GO:0008202	Steroid metabolic process	2.40E‐09	GO:0006082	Organic acid metabolic process	4.10E‐08
	GO:0044281	Small molecule metabolic process	3.90E‐09	GO:0008202	Steroid metabolic process	2.30E‐07
	GO:0016125	Sterol metabolic process	1.90E‐08	GO:0016125	Sterol metabolic process	8.40E‐07
	GO:1901615	Organic hydroxy compound metabolic process	1.40E‐07	GO:0003333	Amino acid transmembrane transport	1.10E‐06
	GO:1901617	Organic hydroxy compound biosynthetic process	1.70E‐07	GO:1901615	Organic hydroxy compound metabolic process	2.50E‐06
	GO:0006066	Alcohol metabolic process	1.90E‐07	GO:0072348	Sulfur compound transport	3.20E‐06

Note

For full table, see File S7.

Upregulation in response to heat stress in both populations was generally characterized by enrichment in small molecule metabolic processes (e.g., GO:0006082 organic acid metabolic process, GO:0044281 small molecule metabolic process, GO: organonitrogen compound biosynthetic process) and immune response (e.g., GO:0002526 acute inflammatory response, GO:0006979 response to oxidative stress, GO:0006956 complement activation). Downregulated biological processes included carbohydrate metabolism (e.g., GO:0006003 fructose 2,6‐bisphosphate metabolic process, GO:0045913 positive regulation of carbohydrate metabolism), While the most significantly enriched processes in the VA population also included transmembrane transport (e.g., GO:0003333 amino acid transmembrane transport, GO:1903825 organic acid transmembrane transport).

Amplification efficiencies of the target genes and the internal control (18S rRNA) ranged from 1.80 to 2.13. Log2 fold changes obtained from RT‐qPCR showed a strong correlation with RNA‐seq results (Pearson's *r* = .995, *p* < .05, Figure S5).

## 4 DISCUSSION

Transcriptomic studies of fish populations originating from contrasting temperature regimes have shown distinct responses to chronic thermal stress, but little is known about the effects of acute thermal stress. We conducted RNA‐seq on two genetically distinct and physiologically divergent populations of spotted seatrout exposed to acute temperature stress and de novo‐assembled the first liver transcriptome for this species. Based on this transcriptome, we compared the molecular mechanisms underlying the response to temperature stress between the two populations. The putatively cold‐adapted northern population showed greater transcriptional response to heat stress, while the putatively warm‐adapted southern population showed greater transcriptional response to cold stress. KEGG pathway and GO enrichment analyses of DEGs revealed both common and unique pathways/biological processes in both populations in response to temperature stress. Log2 fold change of a subset of genes between RT‐qPCR and RNA‐seq were highly consistent, validating the accuracy of the RNA‐seq results.

A high‐quality transcriptome is essential for differential expression analyses and functional annotation (Grabherr et al., 2011). In general, a longer N50 indicates a better assembly. The N50 for our liver transcriptome was 3,121 base pairs (bp), much longer than similar de novo‐assembled transcriptomes for other fishes, including Yellow perch *Perca falvescens* (1,066 bp; Li et al., 2017), Spotted rainbowfish *Melanotaenia duboulayi* (1,856 bp; Smith et al., 2013), and Patagonian toothfish *Dissostichus eleginoides* (1,434 bp; Touma et al., 2019). The percentage of the BUSCO gene content present in the assembled transcriptome (87.4%) was higher than the gene content recovered for the European sardine *Sardina pilchardus* (82.1%; Machado et al., 2018), Patagonian toothfish *Dissostichus eleginoides* (78.92%; Touma et al., 2019), and Clown anemonefish *Amphiprion percula* (85.4%; Maytin et al., 2018). The high‐quality transcriptome can be attributed to the use of high‐quality samples, a combination of assemblers, and a range of k‐mers, as well as the subsequent redundancy‐reducing step. Future transcriptomic studies of spotted seatrout that include new tissue types will likely discover genes that were not expressed in the liver and result in a more comprehensive transcriptome for spotted seatrout.

In comparative studies of populations or closely related species, a larger transcriptomic response can either indicate a general stress response or physiological adaptation (Narum & Campbell, 2015; Veilleux et al., 2018). Thus, transcriptomic studies should be viewed within an ecological and physiological context (DeBiasse & Kelly, 2016). The VA spotted seatrout had a larger range of metabolic plasticity than the SC fish, as well as a higher standard metabolic rate (minimum oxygen consumption rate) between 5 and 30°C (Song et al., 2019). Thus, the larger transcriptional response observed in the southern population at 5°C as compared to the northern population can be interpreted as greater physiological stress. Similarly, a larger transcriptional response by the northern population at 30°C indicates that this population experienced greater physiological stress than the southern population when temperatures were elevated, likely due to a mismatch between oxygen demand (high SMR) and oxygen supply (low dissolved oxygen) (Pörtner & Knust, 2007).

Functional annotation of the spotted seatrout transcriptome also suggested that the southern population experienced greater physiological stress at low temperature, while the northern population experienced greater physiological stress at high temperature. Only the northern population induced genes in the apoptosis pathway (upregulation of proto‐oncogene c‐Fos, cytochrome C, and diablo) in response to heat stress based on KEGG results. The northern population also showed an enrichment in upregulated genes in the response to oxidative stress (GO:0006979), which is predicted because aquatic breathers have increasing difficulty acquiring sufficient oxygen as waters warm, especially those species and populations with high metabolic demands (Heise, 2006; Pörtner & Peck, 2010).

The larger number of DEGs under acute cold stress as compared to actuate heat stress could be an artifact of the endpoint temperature chosen for this study. Ellis et al. (2017) conducted cold tolerance experiments on adult spotted seatrout in North Carolina and found that spotted seatrout could tolerate water temperatures to 5°C for up to five days, after which mortality increased rapidly, and a study by Anweiler et al. (2014) found 91% mortality in juvenile spotted seatrout held at 4.25°C for five days, suggesting that this is the lower limit of temperature tolerance. Thus, 5°C was chosen as the endpoint of the cold stress experiments to elicit a strong physiological stress response. McDonald et al. (2013) reported the critical thermal maximum (temperature at which 50% of the fish die) of juvenile spotted seatrout in Texas, which is near the southern end of the distribution, was around 39°C. Here, 30°C was chosen as a conservative maximum temperature limit for spotted seatrout because this is approximately what natural populations in VA and SC experience in summer. These data suggest that 5°C is closer to spotted seatrout's lower thermal tolerance threshold than 30°C is to its upper tolerance threshold. Future studies aiming to elicit an even stronger physiological stress response to heat in spotted seatrout should choose an endpoint temperature above 30°C.

A higher number of upregulated transcripts compared with downregulated transcripts were discovered in all treatments for both groups. This finding agrees with a study using zebrafish larvae that found severe cold stress at 12°C induced 1,431 upregulated genes compared with 399 downregulated genes (Long et al., 2013). In common killifish, cold acclimation at 5°C induced 5,460 upregulated genes compared with 1,746 downregulated genes in muscle transcriptomes (Healy et al., 2017). This trend is predicted because low temperature depresses the rate of biochemical processes, and increased expression can compensate for this kinetic restraint (Currie & Schulte, 2013). However, there are exceptions; in orange‐spotted grouper (*Epinephelus coioides*), there were 2,093 upregulated genes and 3,812 downregulated genes under cold stress (Sun et al., 2019). This might reflect different physiological strategies among species in coping with low temperatures. Spotted seatrout, zebrafish, and killifish all live in shallow systems where water temperatures undergo large daily and seasonal fluctuations. Coupled with a limited ability to migrate, these species have to turn up their transcriptional machinery to survive periods of low water temperature. In contrast, orange‐spotted grouper experience more stable ambient temperature in the open ocean and therefore may have evolved an opposite strategy by turning down gene expression to conserve energy during cold periods. Transcriptomic studies of strictly marine teleostean species are limited, and more research is needed to understand the impact of acute temperature stress (Logan & Buckley, 2015; Oomen & Hutchings, 2017).

We found 20 shared DEGs in both cold and heat stress in both populations (Figures S2 and S3). These genes most likely play important roles in the cellular stress response (CSR). Seven out of the 20 genes were annotated, and associations included the heat‐shock protein 90‐alpha (*hs90A*), apolipoprotein (*apom*), and haptoglobin (*hpt*). Heat‐shock proteins are a group of well‐studied gene families, which act as generic molecular chaperones to help maintain protein integrity under a range of stressors such as temperature, salinity, and disease (Iwama et al., 2001). Apolipoproteins bind to lipids and play a major role in lipid transport and have been shown to be a part of the innate immunity in fishes (Causey et al., 2018; Concha et al., 2004; Pereiro et al., 2012). *Hpt* is one of the acute‐phase stress proteins synthesized by the liver (Cordero et al., 2017; Windisch et al., 2014) and can bind to hemoglobin in the plasma and reduce oxidative stress (Alayash, 2011). While most of the 20 genes showed similar levels of differential expression between VA and SC (Figures S2 and S3), *hpt* showed the largest differences in log2 fold change (Figure S4, cold stress: 10 versus. 3, heat stress: 10 versus. 5.5). This substantial difference was in part due to the low average expression level of *hpt* in SC controls. We suggest that *hpt* warrants further investigation to confirm this finding, and if the results are repeatable, it would be an excellent genetic marker for estimating population‐level temperature stress in spotted seatrout. Unfortunately, 13 out of the 20 generic stress genes did not return a BLAST hit, highlighting the problem with poor functional annotation in nonmodel organisms (Pavey et al., 2012).

### 4.1 Shared KEGG pathways

A few shared molecular pathways between the northern and southern populations accounted for a disproportionately large number of DEGs (Table 2). Metabolic pathways accounted for the most DEGs. The MAPK signaling pathway was one of the top pathways observed in response to heat stress in spotted seatrout and is one of the hallmarks of CSR in other organisms (Kültz, 2004). MAPKs are kinases that are involved in protein phosphorylation cascades in order to regulate the expression of many downstream targets (Cowan & Storey, 2003; Huang et al., 2017). This pathway has been shown to be particularly important in modulating gene expression in gill epithelium cells in killifish during osmoregulation (Kültz & Avila, 2001) and in the swim bladders of channel catfish (*Ictalurus punctatus*) in response to low dissolved oxygen (Yang et al., 2018). Under cold stress, the FoxO signaling pathway was a top pathway in both populations. Similar to the MAPK pathway, the FoxO signaling pathway can effect global transcriptomic change via a group of transcription factors that target genes involved in apoptosis, oxidative stress resistance, and cell cycle control (Puig & Mattila, 2011; Ronnebaum & Patterson, 2010).

### 4.2 Population‐specific genes and KEGG pathways

In response to cold stress, the northern population downregulated acyl‐coenzyme A thioesterase (*acot*) in the fatty acid elongation (lco00062) and biosynthesis of unsaturated fatty acid (lco01040) pathways. *Acot* plays a critical role in fatty acid metabolism and ATP generation (Tillander et al., 2017). This suggests that fat metabolism is suppressed, at least in the short term, in response to cold stress. In addition, genes involved in protein synthesis and transport were upregulated in pathways such as ribosome biogenesis in eukaryotes (lco03008) and protein export (lco03060). These genes included ribonuclease P protein subunit (*pop4*), U4/U6 small nuclear ribonucleoprotein (*snu13*), and transport protein subunit alpha (*sec61a*). 26S proteasome regulatory subunit T3 (*psmc4*) in the proteasome (lco03050) pathway is also upregulated. The proteasome is a large molecular complex where protein degradation occurs (Glickman & Ciechanover, 2002). Protein synthesis, export, and degradation are all energetically expensive processes requiring ATP. The upregulation of genes in these pathways suggests the northern population is capable of producing sufficient ATP to meet energy requirements at low temperature.

Under acute cold stress, the southern population was found to uniquely upregulate long‐chain acyl‐CoA synthetase (*acsl*) and long‐chain fatty acid–CoA ligase (*acsbg*) in the fatty acid biosynthesis (lco00061) and fatty acid metabolism (lco01212) pathways. Both genes play critical roles in the fatty acids oxidation (Cheng et al., 2017). The large subunit ribosomal protein L23Ae (*rp‐l23ae*) in the ribosome (lco03010) pathway is upregulated, suggesting enhanced protein production. However, genes in the protein degradation pathway, lysosome (lco04142), are downregulated: deoxyribonuclease II (*dnase2*), lysosomal alpha‐glucosidase (*gaa*), and acid ceramidase (*asah1*). Lysosomes are organelles in eukaryotic cells containing hydrolytic enzymes that degrade biomolecules (Levine & Klionsky, 2004). A mismatch between protein synthesis and protein degradation may suggest the increased accumulation of misfolded proteins in the cell and can lead to apoptosis (Fribley et al., 2009).

The northern and southern populations of spotted seatrout also showed distinct responses to heat stress. Under acute heat stress, the northern population uniquely upregulated heat shock 70 kDa protein (*hspa5*), a member of the heat‐shock protein 70 family (Roberts et al., 2010). Acetyl‐CoA synthetase (*acss1*) was upregulated in the pyruvate metabolism (lco00620) and carbon metabolism (lco01200) pathways. *Acss1* catalyzes the reaction that produces the raw material, acetyl‐CoA, for the citric acid cycle (aka. tricarboxylic acid cycle) in order to produce ATP. However, this pathway is only activated when cells have depleted their normal carbon source (pyruvate) (Wolfe, 2005). Isocitrate dehydrogenase (*idh1*), which is involved in the citric acid cycle, was also uniquely upregulated. The higher metabolic demands of the northern fish observed in previous physiology studies (Song et al., 2019) may have quickly exhausted pyruvate, and the upregulation of *acss1* serves as a short‐term solution to supply acetyl‐CoA to generate ATP. In addition, genes in the N‐glycan biosynthesis (lco00510) pathway were upregulated: alpha‐1,2‐mannosyltransferase (*alg9*) and mannosyl‐oligosaccharide glucosidase (*mogs*). These enzymes are involved in N‐linked glycosylation (addition of oligosaccharides to proteins) and play important roles in protein stability and cell signaling (Sinclair & Elliott, 2005). GO analysis also identified glycosylation‐related processes to be enriched in VA fish (Table 3. GO:0006487. GO:0009101, GO:0009100).

Similarly, under heat stress, the southern population uniquely upregulated heat‐shock cognate 71 kDa protein (*hsc71*). *Hsc71* belongs to the same protein family as *hspa5*, which was uniquely upregulated in the northern population, and performs similar functions; however, *hsc71* is known to be constitutively expressed in unstressed cells and *hspa5* is only upregulated during stress (Goldfarb et al., 2006; Roberts et al., 2010). *Hsc71* was also found to be inducible under heat stress in gill tissues of *Gillichthys mirabili* (Buckley, 2006). RAC serine/threonine‐protein kinase (*akt*) is downregulated in the following pathways: mTOR signaling pathway (lco04150), insulin signaling pathway (lco04910), and apelin signaling pathway (lco04371). G1/S‐specific cyclin‐D2 (*ccnd2*) was downregulated in the hedgehog signaling pathway (lco04340). Both *akt* and *ccnd2* play a role in cell proliferation and the cell cycle (Katoh & Katoh, 2009; Manning & Cantley, 2007); thus, their downregulation may indicate heat stress also induces cell growth arrest in the southern population.

This study, which examined transcriptomic responses to acute temperature stress in spotted seatrout, provided novel insights into stress response. For example, cytoskeletal re‐organization is among the significantly enriched biological processes in studies, which applied a chronic temperature stress (Healy et al., 2017; Metzger & Schulte, 2017; Narum & Campbell, 2015; Newton et al., 2013; Sun et al., 2019); however, we did not find this process to play a significant role under acute temperature stress. This finding is consistent with a recent study that applied an acute heat stress protocol (2°C/hr, 20–32°C) to small yellow croaker, *Larimichthys polyactis* (Chu et al., 2020). This discrepancy suggests that gene expression underlying transcriptomic plasticity may act on different timescales (Metzger & Schulte, 2017) and requires more studies to understand the commonalities of response to acute temperature stress in fishes.

The fish used in this study were part of an ongoing respirometry study, and there are potential caveats that should be considered when interpreting the results. One limitation concerns the origin of the SC control samples, which were not wild‐caught, but instead were the F1 generation of wild parents. Although the controls were from the F1 generation, the rearing tanks received water directly from Charleston Harbor, and thus experienced the same natural temperature fluctuations as their wild counterparts. Admittedly, other aspects of being in captivity may have altered gene expression, but all fish in the treatment group experienced acclimation in this same environment. Therefore, we contend that the fish in the control group, although not optimal, were appropriate for this study. Secondly, gene expression patterns are known to be affected by seasonality (Tian et al., 2019) and sex (Selmoni et al., 2020). Due to logistic difficulties, all fish were captured in fall/winter (October/November) with the exception of the SC fish used in the heat stress experiments, which were captured in spring (March) (Table S1). Despite the evidence that eurythermal fishes require much less time (days to a week) to acclimate than more stenothermal fishes (weeks to months) (Healy & Schulte, 2012), it is not well understood how differences in acclimation times affect gene expression in eurythermal fishes. Finally, this study used a mix of sexes based on visual examination of gonads. Thus, it is possible that there were sex‐specific transcriptomic responses to temperature stress that were unaccounted for in this study.

In summary, we present evidence that differences in thermal tolerance, as reflected by differential gene expression, contribute to the population‐level divergence observed in spotted seatrout from the U.S. South Atlantic and provide mechanistic insights into physiological responses to acute temperature stress. Our results indicate that the northern population shows transcriptional signatures consistent with cold adaptation, yet they may be more vulnerable to acute heat waves than the southern population. Likewise, the transcriptomic profiles of the southern fish indicate that they have a greater stress response than northern fish when exposed to cold temperatures. These differences may play a role in maintaining the observed genetic differences between these two populations. The liver transcriptome represents a valuable resource for future genetic monitoring studies. Candidate genes (e.g., hpt, acot, hsc71, hspa*5)* identified in this study should be functionally validated or screened more broadly across the species' range to verify their ecological importance. Furthermore, genomic‐level and cross‐generational investigations would also complement this study by discovering genes under selection and improve our understanding of adaptive evolution in general.

## CONFLICT OF INTEREST

None declared.

## AUTHOR CONTRIBUTION

**Jingwei Song**: Conceptualization (equal); Data curation (lead); Formal analysis (lead); Funding acquisition (equal); Investigation (lead); Methodology (lead); Project administration (equal); Resources (equal); Software (lead); Validation (lead); Visualization (lead); Writing‐original draft (lead); Writing‐review & editing (equal). **Jan Renee McDowell**: Conceptualization (equal); Funding acquisition (lead); Project administration (equal); Resources (equal); Supervision (supporting); Validation (supporting); Writing‐review & editing (equal).

## Supporting information

Appendix S1

Appendix S2

Legends S1

## Data Availability

RNA‐seq sequences are available on NCBI SRA database with project accession No. PRJNA649515. All supplemental materials including the final transcriptome assembly and Trinotate annotation report have been uploaded on William & Mary ScholarWorks (https://doi.org/10.25773/yp0v‐zc62).
