# Supplementary material for: Comparative transcriptomics of spotted seatrout (Cynoscion nebulosus) populations to cold and heat stress
Source: Ecol Evol. 2020 Dec 28;11(3):1352–67. doi: 10.1002/ece3.7138 (PMC7863673; doi:10.1002/ece3.7138)
Supplement: Supplementary file 1 — Appendix S1 [file ECE3-11-1352-s001.docx]

**Appendix.**

Table S1. Spotted seatrout (*Cynosion nenulosus*) samples used in this study. All fish were adults and reproductively inactive based on visual examination of gonads when liver tissue was collected. F1 generation from wild caught parents (JS21 to JS25) were used to supplement SC samples. Prior to acclimation start for fish in the heat stress group (JS11-JS20), they were held at the same condition as the fish in the cold stress group.

| RNA-seq ID | Collection date | Location | Coordinates | Group | Acclimation started | Experiment date | Acclimation temp (°C) | Sampling temp (°C) | Total Length (mm) |
| --- | --- | --- | --- | --- | --- | --- | --- | --- | --- |
| JS01 | 11/23/2017 | SC | 32.753055, -79.896670 | cold stress | 11/23/2017 | 1/23/2018 | 15 | 5 | 290 |
| JS02 | 11/23/2017 | SC | 32.753055, -79.896670 | cold stress | 11/23/2017 | 2/15/2018 | 15 | 5 | 370 |
| JS03 | 11/23/2017 | SC | 32.753055, -79.896670 | cold stress | 11/23/2017 | 2/19/2018 | 15 | 5 | 350 |
| JS04 | 11/23/2017 | SC | 32.753055, -79.896670 | cold stress | 11/23/2017 | 2/23/2018 | 15 | 5 | 400 |
| JS05 | 11/23/2017 | SC | 32.753055, -79.896670 | cold stress | 11/23/2017 | 2/26/2018 | 15 | 5 | 370 |
| JS06 | 11/6/2017 | VA | 37.732985, -76.408968 | cold stress | 11/6/2017 | 1/23/2018 | 15 | 5 | 300 |
| JS07 | 11/6/2017 | VA | 37.732985, -76.408968 | cold stress | 11/6/2017 | 2/19/2018 | 15 | 5 | 300 |
| JS08 | 11/6/2017 | VA | 37.732985, -76.408968 | cold stress | 11/6/2017 | 2/19/2018 | 15 | 5 | 290 |
| JS09 | 11/6/2017 | VA | 37.732985, -76.408968 | cold stress | 11/6/2017 | 2/23/2018 | 15 | 5 | 300 |
| JS10 | 11/6/2017 | VA | 37.732985, -76.408968 | cold stress | 11/6/2017 | 2/26/2018 | 15 | 5 | 300 |
| JS11 | 3/4/2018 | SC | 32.753055, -79.896670 | heat stress | 6/6/2018 | 7/6/2018 | 20 | 30 | 350 |
| JS12 | 3/4/2018 | SC | 32.753055, -79.896670 | heat stress | 6/6/2018 | 7/10/2018 | 20 | 30 | 450 |
| JS13 | 3/4/2018 | SC | 32.753055, -79.896670 | heat stress | 6/6/2018 | 7/13/2018 | 20 | 30 | 380 |
| JS14 | 3/4/2018 | SC | 32.753055, -79.896670 | heat stress | 6/6/2018 | 7/18/2018 | 20 | 30 | 395 |
| JS15 | 3/4/2018 | SC | 32.753055, -79.896670 | heat stress | 6/6/2018 | 7/28/2018 | 20 | 30 | 370 |
| JS16 | 11/6/2017 | VA | 37.732985, -76.408968 | heat stress | 6/6/2018 | 7/6/2018 | 20 | 30 | 300 |
| JS17 | 11/6/2017 | VA | 37.732985, -76.408968 | heat stress | 6/6/2018 | 7/22/2018 | 20 | 30 | 300 |
| JS18 | 11/6/2017 | VA | 37.732985, -76.408968 | heat stress | 6/6/2018 | 8/5/2018 | 20 | 30 | 360 |
| JS19 | 11/6/2017 | VA | 37.732985, -76.408968 | heat stress | 6/6/2018 | 8/10/2018 | 20 | 30 | 340 |
| JS20 | 11/6/2017 | VA | 37.732985, -76.408968 | heat stress | 6/6/2018 | 8/20/2018 | 20 | 30 | 320 |
| JS21 | 10/23/2016 | SC | 32.753055, -79.896670 | control | 10/25/2016 | 3/8/2017 | 15 | 15 | ≥250 |
| JS22 | 10/23/2016 | SC | 32.753055, -79.896670 | control | 10/25/2016 | 4/23/2017 | 15 | 15 | ≥250 |
| JS23 | 10/23/2016 | SC | 32.753055, -79.896670 | control | 10/25/2016 | 4/23/2017 | 15 | 15 | ≥250 |
| JS24 | 10/23/2016 | SC | 32.753055, -79.896670 | control | 10/25/2016 | 4/23/2017 | 15 | 15 | ≥250 |
| JS25 | 10/23/2016 | SC | 32.753055, -79.896670 | control | 10/25/2016 | 4/23/2017 | 15 | 15 | ≥250 |
| JS26 | 11/2/2016 | VA | 37.732985, -76.408968 | control | 11/2/2016 | 3/8/2017 | 15 | 15 | ≥300 |
| JS27 | 11/2/2016 | VA | 37.732985, -76.408968 | control | 11/2/2016 | 3/8/2017 | 15 | 15 | ≥300 |
| JS28 | 11/2/2016 | VA | 37.732985, -76.408968 | control | 11/2/2016 | 3/8/2017 | 15 | 15 | ≥300 |
| JS29 | 11/2/2016 | VA | 37.732985, -76.408968 | control | 11/2/2016 | 3/8/2017 | 15 | 15 | ≥300 |
| JS30 | 11/2/2016 | VA | 37.732985, -76.408968 | control | 11/2/2016 | 3/8/2017 | 15 | 15 | ≥300 |

Table S2. Primers used in RT-qPCR. 18S primers were obtained from Brewton et al. (2013). The rest were designed based on de novo assembled transcripts for this study.

| Oligo sequence (5' to 3') | Oligo name |
| --- | --- |
| CCAACGAGCGCTGACCTCCG | 18S_F |
| GAGTCACCAAAGCGGCCGGG | 18S_R |
| ACAAAGCTGGATTTGGCAGC | mic2_F |
| CCGATTCTGGACCCACAGAG | mic2_R |
| GTTCAAACACGCCACCTGAG | apo2_F |
| CTACGTCCACACGTCCTGTC | apo2_R |
| GGCACGGAATTCAAGCTGAC | hsp4_F |
| GGACCCGTAACCCAGATGAC | hsp4_R |
| TGGTGGTCACATCATCAGGC | ped2_R |
| TCGGTTCGGTCAAAGTGGAG | ped2_R |
| TGGATCAGTGAGCAAAGGGC | lec2_F |
| TCTGGACGTGGACATGTGAG | lec2_R |
| ATGGAAGGGGTCCACTTGAG | cea2_F |
| CCTGCTTGACGAGCTGTACC | cea2_R |

Table S3. Quality metrics of the spotted seatrout liver transcriptome based on QUAST v4.6.3 report.

| # contigs (>= 0 bp) | 37398 |
| --- | --- |
| # contigs (>= 1000 bp) | 14669 |
| # contigs (>= 5000 bp) | 1674 |
| # contigs (>= 10000 bp) | 123 |
| # contigs (>= 25000 bp) | 0 |
| # contigs (>= 50000 bp) | 0 |
| Total length (>= 0 bp) | 51904107 |
| Total length (>= 1000 bp) | 42353733 |
| Total length (>= 5000 bp) | 11607548 |
| Total length (>= 10000 bp) | 1547531 |
| Total length (>= 25000 bp) | 0 |
| Total length (>= 50000 bp) | 0 |
| # contigs | 21316 |
| Largest contig | 24504 |
| Total length | 47027336 |
| GC (%) | 48.98 |
| N50 | 3121 |
| N75 | 1840 |
| L50 | 4735 |
| L75 | 9620 |
| # N's per 100 kbp | 392.15 |

Figure S1. Plot of principal component analysis based on gene expression values for all 30 samples, color-coded by groups. VA=northern population, SC=southern population, c=cold stress, h=heat stress, ctrl=control.


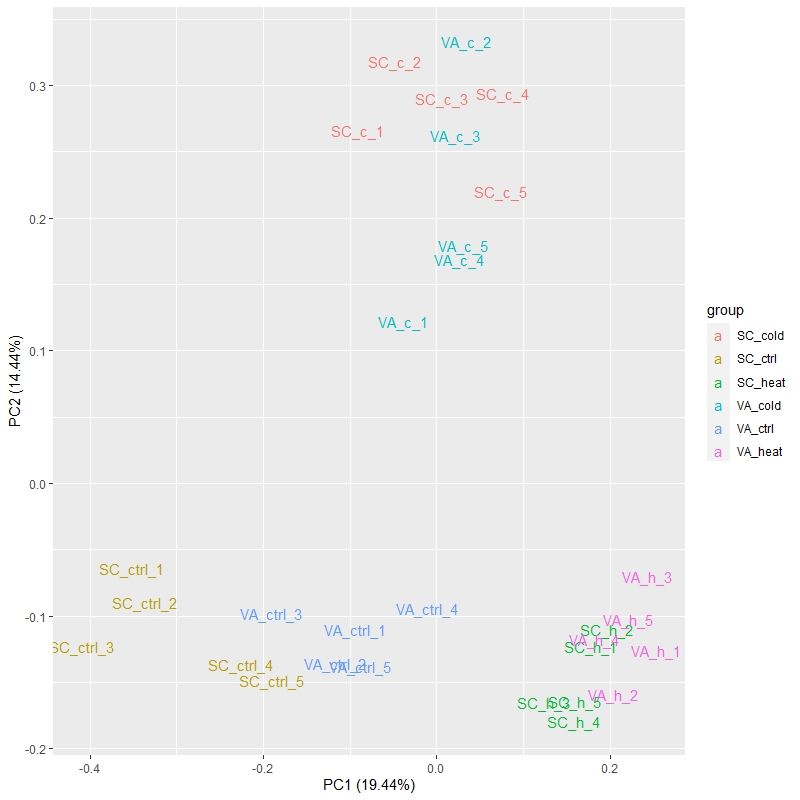


Figure S2. Log2 fold change values for the 20 shared differentially expressed genes (DEGs) that responded to both cold and heat stress in both populations (values for cold stress only).

Figure S3. Log2 fold change values for the 20 shared differentially expressed genes (DEGs) that responded to both cold and heat stress in both populations (values for heat stress only).

Figure S4. Volcano plots showing differential expression in spotted seatrout subjected to cold stress. Y axis shows -log_10_(FDR-corrected *p*-value) and x axis shows log2 fold change. Top, South Carolina (SC), bottom, Virginia (VA). Red dots are significant differentially expressed genes (DEG), black dots are non-significant DEGs. Haptoglobin (hpt) is indicated by an arrow in SC. Hpt is not clearly visible in the bottom plot and therefore not shown.


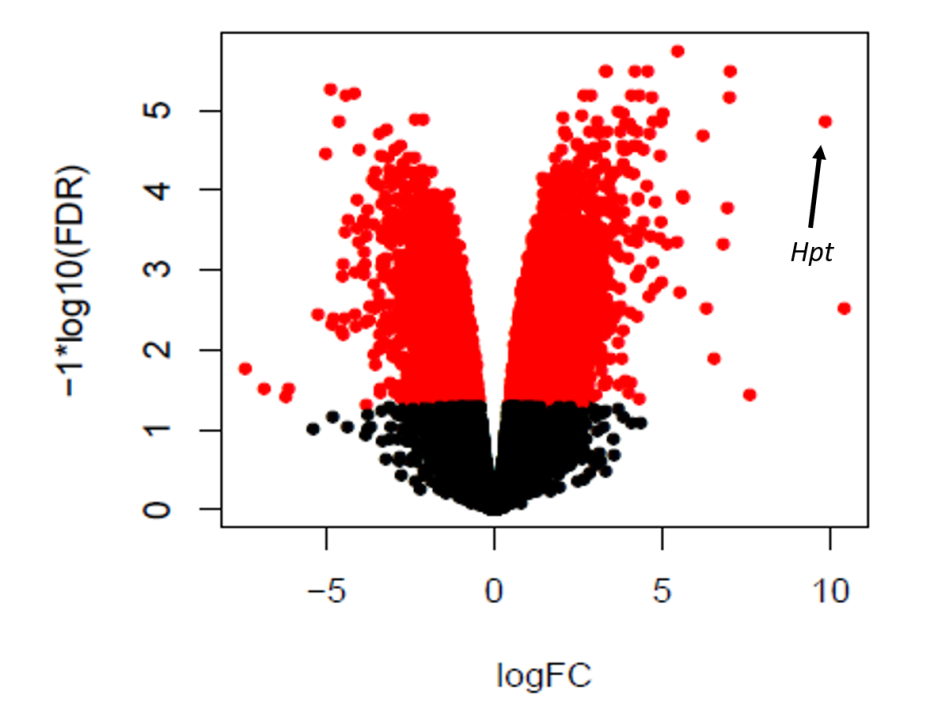


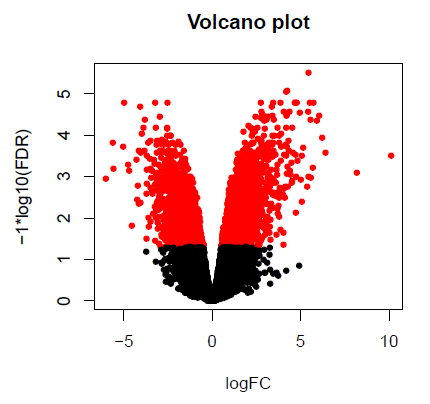


Figure S5. Comparisons of log_2_ fold change between RNA-seq and RT-qPCR results. Primer/sample pairs are as follows: mic2, apo2 (JS01-05 and JS21-25); hsp4, ped2 (JS06-10 and JS26-30); lec2, cea2 (JS11-15 and JS21-25)
