# Supplementary material for: Comparative transcriptomics of spotted seatrout (Cynoscion nebulosus) populations to cold and heat stress
Source: Ecol Evol. 2020 Dec 28;11(3):1352–67. doi: 10.1002/ece3.7138 (PMC7863673; doi:10.1002/ece3.7138)
Supplement: Supplementary file 3 — Legends S1 [file ECE3-11-1352-s003.docx]

**Legends**

**Supplemental Information (doi: 10.25773/yp0v-zc62)**

SuppInfo_S1. Spotted seatrout liver transcriptome in FASTA format.

SuppInfo_S2. Functional annotation report from Trinotate v3.1.1 for the spotted seatrout liver transcriptome.

SuppInfo_S3. Significant common transcripts responsive to both cold and heat stress and in both the southern and northern populations.

SuppInfo_S4. Transcript names showing significant differential expression to temperature stress, separated by populations.

SuppInfo_S5. Transcripts quantification matrix for all samples, generated by kallisto v0.43.1.

SuppInfo_S6. Names of common and unique KEGG molecular pathways between and within populations.

SuppInfo_S7. Significant biological processes based on Gene Ontology enrichment analysis of differentially expressed genes (DEGs).
